# Supplementary material for: Social support and technophobia in older patients with coronary heart disease: The mediating roles of eHealth literacy and healthcare technology self-efficacy
Source: PLoS One. 2025 Jun 4;20(6):e0325202. doi: 10.1371/journal.pone.0325202 (PMC12136290; doi:10.1371/journal.pone.0325202)
Supplement: Attachment — Submitted filename: Response to Reviewers.docx [file pone.0325202.s003.docx]

November 27, 2024

Dear Dr. Saeideh Valizadeh-Haghi,

RE: Manuscript ID PONE-D-24-40957

Thank you very much for giving us the opportunity to revise our manuscript entitled “Social support and technophobia in older patients with coronary heart disease: The mediating roles of eHealth literacy and healthcare technology self-efficacy”. We appreciate the valuable and constructive comments and suggestions from the editor, reviewers, and statisticians on this manuscript. Their insightful comments and suggestions have undoubtedly contributed to the improvement of our manuscript.

We have deliberately considered all comments and suggestions and have revised our manuscript accordingly. Please find our point-by-point responses in the response letter.

With the commitment and approval of all authors, we hereby submit our revised manuscript. We guarantee that neither the text nor the abstract of this paper has been published elsewhere in any language. We have no conflicts of interest to declare.

Thank you once again for your time, consideration, and the opportunity to submit our revised manuscript. We hope that the revised manuscript meets your requirements for publication in PLOS ONE.

Financial disclosure of this study:

1. AW

General Program of the Natural Science Foundation of Shandong Province

grant number: ZR2023MG071

URL: http://kjt.shandong.gov.cn/

The funders had no role in study design, data collection and analysis, decision to publish, or preparation of the manuscript.

2. DH

Youth Fund of the Natural Science Foundation of Shandong Province

grant number: ZR2023QG027

URL: http://kjt.shandong.gov.cn/

The funders had no role in study design, data collection and analysis, decision to publish, or preparation of the manuscript.

Yours sincerely,

Aimin Wang

Institution: College of Nursing, Qingdao University

E-mail: wam@qdu.edu.cn

The point-by-point replies to all comments have been provided as follows:

**Journal requirements:**

1. Please ensure that your manuscript meets PLOS ONE's style requirements, including those for file naming. The PLOS ONE style templates can be found at

https://journals.plos.org/plosone/s/file?id=wjVg/PLOSOne_formatting_sample_main_body.pdf and

https://journals.plos.org/plosone/s/file?id=ba62/PLOSOne_formatting_sample_title_authors_affiliations.pdf

Response: Thank you for your suggestions, we have scrutinized the formatting of the manuscript and identified a few minor issues which have been corrected in the revised manuscript. Specific amendments are as follows: add a symbol legend for the author's signature “^1^” [Page1, Line 9,13]; Change the first level heading “Abstract” “Study limitations” to font size 18. [Page18, Line 413]

2. Please include a caption for figure 1.

Response: Thank you for your careful review, we have added the caption for Figure 1 in the revised version.

Here is the revised version: “Fig 1. The chain mediation model of eHealth literacy and healthcare technology self-efficacy between social support and technophobia.” [Page12, Line 281-282]

3. Please include a caption for table 4.

Response: Thank you for your comment. We sincerely apologize for the oversight of Table 4 in the previous version, which has now been included in full in the revised version. [see revised Page 14, Line 301-308]

4. Thank you for stating the following financial disclosure:

[AW

Upper-level Project of the Natural Science Foundation of Shandong Province

grant number: ZR2023MG071

URL: http://kjt.shandong.gov.cn/

the founder gave a financial support in paper submission.

DH

Youth Fund of the Natural Science Foundation of Shandong Province

grant number: ZR2023QG027

URL: http://kjt.shandong.gov.cn/

the founder gave a financial support in paper submission.].

Please state what role the funders took in the study. If the funders had no role, please state: “The funders had no role in study design, data collection and analysis, decision to publish, or preparation of the manuscript.”

If this statement is not correct you must amend it as needed.

Please include this amended Role of Funder statement in your cover letter; we will change the online submission form on your behalf.

Response: Thank you for your comment. We apologize for using the wrong statement, and we have submitted the correct financial disclosure statement in the cover letter.

5. Please include a copy of Table 4 which you refer to in your text on page 11.

Response: Thank you very much for your careful review. We sincerely apologize for the oversight of Table 4 in the previous version, which has now been included in full in the revised version. [see revised Page 14, Line 300-307]

6. Please review your reference list to ensure that it is complete and correct. If you have cited papers that have been retracted, please include the rationale for doing so in the manuscript text, or remove these references and replace them with relevant current references. Any changes to the reference list should be mentioned in the rebuttal letter that accompanies your revised manuscript. If you need to cite a retracted article, indicate the article’s retracted status in the References list and also include a citation and full reference for the retraction notice.

Response: Thank you for your request. We have added 13 references (Refs. 1, 4-6, 32, 34, 37, 39, 40, 41, 43-45) and deleted 2 references (Refs. 38, 42) because of changes to the article. Excluding additions and deletions, we have double-checked the list of references. We have added page numbers to references to make them complete (Refs. 18), and we have changed the names of journals that were not standardized in the references (Refs. 30, 46), as well as changed the format of the author's name in one reference (Refs. 29). We have not cited the retracted references. However, we are open to any further feedback or specific areas that may need further refinement.

**Reviewer(s) comments:**

The submission is well-written, well-organized, and features an interesting topic. Here are some suggestions to enhance clarity and readability.

Overall response to Reviewer: Thank you for spending time reviewing our manuscript and providing us with a list of constructive comments.

Introduction

The Introduction is comprehensive and well-structured, providing a clear overview of the topic and the rationale for the study. Here are some suggestions to enhance clarity and readability:

1. The opening sentence is crucial in the introduction; the researchers should consider starting with a more engaging sentence to capture the reader’s attention. For example: “coronary heart disease (CHD) remains a leading cause of morbidity and mortality worldwide, particularly among the elderly.”

Response: Thank you very much for your advice. Your suggestion was so helpful that we have changed the first two sentences of the introduction in the revised version.

Here is the revised version: “Coronary heart disease (CHD) remains a leading cause of mortality worldwide, particularly among the elderly[1]. In China, the mortality rate of cardiovascular diseases accounts for the first cause of death, with a prevalence of CHD among individuals aged 60 and older reaching 27.8%[2].” [Page 2, Line 49-51]

2. The submission needs minor revision for smoother transitions between paragraphs to ensure a cohesive flow of ideas.

Response: Thank you very much for this suggestion. We have scrutinized the logic from paragraph to paragraph and added some transition sentences to make the logic flow better in the revised version.

Here is the revised version:

(1) “In this context, technophobia emerges as a key issue.” [ Page 3, Line 81]

(2) “Given its potential to hinder older adults from benefiting from these technologies, identifying the protective factors against technophobia to reduce its impact on this population is essential.” [Page 4, Line 89-91]

(3) “Apart from social support, eHealth literacy is also an essential factor influencing technophobia.” [Page 4, Line 100]

(4) “Additionally, healthcare technology self-efficacy is a key internal protective factor affecting technophobia, playing a critical role in guiding technology use behavior[24]. Defined as an individual’s confidence in using digital healthcare technology, healthcare technology self-efficacy provides a more sensitive measure of an individual's confidence in using digital healthcare technology within a healthcare setting compared to general self-efficacy[25].” [Page 4, Line 107-111]

(5) “Furthermore, eHealth literacy is a protective factor for the self-efficacy of older adults in using health technology[25].” [Page 5, Line 117-119]

3. It is recommended to clarify the existing gap; researchers should succinctly outline the traditional way of accessing medical information and assistance and explain why them are burdensome for healthcare organizations. I mean before or right after this sentence: “Consequently, the traditional way of accessing medical information and assistance burdensome for healthcare organizations.”

Response: Thank you very much for this suggestion. We have explained traditional health care and why it adds to the burden in the revised edition.

Here is the revised version: “With the deepening of aging, the demand for healthcare services among elderly patients with CHD has increased, far exceeding that of other age groups. Traditional healthcare services for CHD are based on outpatient clinics, hospital wards or rehabilitation centers, where healthcare professionals provide disease treatment, medication management, dietary guidance and health education[4]. However, elderly patients with CHD may be reluctant to seek medical care due to the distance from healthcare facilities or the high costs involved, which leads to a higher risk of recurrent events and hospitalizations, ultimately diminishing their chances of survival and quality of life[5]. Additionally, in China, there is an imbalance in the distribution of healthcare resources and a shortage of necessary infrastructure and specialized staff[6]. Consequently, the traditional way of accessing medical information and assistance is burdensome for healthcare organizations.” [Page 3, Line 56-66]

Material and methods

The “Materials and Methods” section is quite comprehensive and well-structured. Here are a few suggestions to improve clarity and readability:

4. The researcher mention that a convenience sampling method was used. It may be useful to briefly discuss the potential limitations of this method, such as selection bias, and how it may affect the generalizability of the findings.

Response: Thank you very much for this suggestion. We have provided a detailed explanation of how the convenience sampling method may lead to selection bias and discussed its potential impact on the results in the study limitations section. Additionally, we also make recommendations for future research in the revised version.

Here is the revised version: “Second, although the participants came from different communities, they were recruited from a single province in China. The convenient sampling method may have introduced selection bias. Because elderly patients with CHD in community outpatient may have milder conditions compared to those in hospital wards, and we excluded individuals in the acute attack period of CHD or with other serious illnesses, this may have led to subtle differences in the results. This limits the generalizability of the results to a broader elderly patient with CHD population. It is recommended that future studies employ probability sampling methods to investigate elderly patients with CHD in various regions and settings.” [Page 18, Line 415-422]

5. The inclusion and exclusion criteria are clearly stated. However, it might be beneficial to justify the exclusion of individuals with acute CHD or other serious diseases, as this could be a significant factor affecting the nuances of your findings.

Response: Thank you very much for your comment. For ethical reasons, we were unable to include patients who are in the acute stage of CHD or suffering from serious illnesses. Therefore, we have added an explanation regarding this limitation in the study limitations section of the study.

Here is the revised version: “Because elderly patients with CHD in community outpatient may have milder conditions compared to those in hospital wards, and we excluded individuals in the acute attack period of CHD or with other serious illnesses, this may have led to subtle differences in the results.” [Page 18, Line 417-420]

6. The list of demographic characteristics is comprehensive. Consider providing a rationale for the choice of these particular variables and how they relate to your study’s aims.

Response: Thank you very much for this suggestion. We selected demographic factors that may affect the outcome variable through a literature review to be analyzed first in univariate analysis and put the statistically significant ones as control variables in the mediation analysis. This approach helps minimize the impact of confounding factors, enabling a clearer understanding of the mediation mechanism and identifying the key factors that play a critical role in the causal pathway. In the revised version, we have explained how demographic factors were selected and supplemented the section on control variables in the statistical analysis.

Here is the revised version:

(1) “Based on a comprehensive review of the literature, we selected demographic factors that may influence the outcome variables and independently developed a questionnaire.” [Page 6, Line 146-147]

(2) “Independent samples t-test or one-way ANOVA was used to compare differences in the demographic characteristics. In the mediation effects analysis that followed, variables that showed significant differences in demographic characteristics were controlled for as covariates.” [Page 8, Line 218-220]

7. The inclusion of Cronbach's alpha values is helpful. It would be useful to provide a short explanation on how these statistics indicate the reliability of the scales used. For example, a Cronbach’s alpha above 0.7 is generally considered acceptable in social science research.

Response: Thank you very much for your suggestion. We have added to the research instrument section how these statistics indicate the scales' reliability in the revised version.

Here is the revised version: “These values are greater than the acceptable value of 0.70, which indicates that the Chinese version of the Technophobia Scale has good reliability.” [Page 6, Line 158-160]

8. Include more details about the development or validation processes of Technophobia and Social Support Measures in the Chinese context if available. This would strengthen the credibility of the scales.

Response: Thank you very much for your suggestion. We have added the reliability of scale validation in the Chinese context to all four scales in the revised version.

Here is the revised version:

(1) “The Chinese version of the Technophobia Scale contains 13 items, 3 dimensions: techno-anxiety, techno-paranoia, and privacy concerns, with responses on a five-point Likert scale from 1 (“strongly disagree”) to 5 (“strongly agree”). The total score ranges from 13 to 65 points, with higher scores representing higher levels of technophobia. The Cronbach’s α coefficient was 0.91 for the total scale and 0.88, 0.83, and 0.75 for the three factors, respectively. These values are greater than the acceptable value of 0.70, which indicates that the Chinese version of the Technophobia Scale has good reliability. The Cronbach’s α of the Technophobia Scale in this study was 0.89.” [Page 6, Line 152-160]

(2) “The SSRS has good reliability and validity with the Cronbach’s α of 0.89 to 0.94.” [Page 6, Line 166-167]

(3) “Cronbach’s α for the Chinese version of eHEALS was 0.91, and the Cronbach’s α for the eHEALS in this study was 0.98.” [Page 7, Line 174-175]

(4) “The Cronbach’s α of the Chinese version of the Healthcare Technology Self-Efficacy Scale was 0.93, the split-half reliability was 0.81, and the re-test reliability after two weeks was 0.89.” [Page 6, Line 183-184]

9. It is good to see ethical approval included. The researcher might consider addressing any specific measures taken to ensure the confidentiality and anonymity of participants.

Response: Thank you very much for your suggestion. In the revised version, we have added specific methods to ensure the confidentiality and anonymity of participants in the data collection section.

Here is the revised version:

(1) “All participants were informed that it was an anonymous survey and they had the right to refuse to participate or withdraw at any time during the study.” [Page 7, Line 192-194]

(1) “All paper questionnaires with the informed consent form were only accessible to the research team to ensure security and confidentiality.” [Page 8, Line 200-202]

10. The process of recruitment and data collection is well described. However, detailing how participants were informed about their rights (e.g., right to withdraw) could strengthen the ethical considerations.

Response: Thank you very much for your suggestion. We have explained that all participants have the right to refuse or withdraw at any time during the study in the revised version.

Here is the revised version: “All participants were informed that it was an anonymous survey and they had the right to refuse to participate or withdraw at any time during the study.” [Page 7, Line 192-194]

11. The researcher mentioned checking completed questionnaires for deficiencies or errors. Elaborating on what specific types of deficiencies or errors were checked could add thoroughness to this section.

Response: Thank you very much for your suggestion. We have added specific types of flaws or errors in the data collection section of the exclusion questionnaire.

Here is the revised version: “The researcher checked the completed questionnaires immediately and asked the participants to provide any missing data. Questionnaires with apparent regularities and logical errors were eliminated, such as a questionnaire with at least a string of more than 10 consecutive identical item responses.” [Page 8, Line 197-200]

Statistical analyses

12. (1) Mentioning the software used is good practice. The researchers may consider briefly explaining why they chose SPSS and AMOS and their appropriateness for their analyses.

(2) The mention of testing for common method bias is excellent. The authors might elaborate on the techniques or specific tests performed in AMOS for clarity.

(3) When discussing the use of the PROCESS macro for mediation analysis, consider including a brief explanation of the significance of mediation analysis, particularly for readers who may not be familiar with it.

Response: Thank you very much for your suggestions. In response to the above three suggestions regarding the data analysis section, we have made careful revisions. First, we provided a detailed explanation of the common method bias technique used in this study, along with the rationale for selecting this method. We then clarified how it was applied in AMOS and the significance of the results. Second, we added a section on univariate analysis, where variables that are meaningful in the univariate analysis will be included as control variables in the mediated effects analysis. Finally, we elaborate on the method used to test the mediation effect in PROCESS and its advantages.

Here is the revised version:

“SPSS 25.0 and AMOS were used for data analysis. Common method bias was tested by AMOS and chained mediated effect analysis was performed using SPSS. In order to improve the rigor of the study, we tested for common method bias first. Common method bias refers to artifactual covariation between a predictor and a valid scale variable because of the same data source or rater, the same measurement environment, the context of the item, and the characteristics of the item itself. Because this study collected data through self-reporting methods, it was possible that there could be an issue with common method bias. Podsakoff et al. recommend introducing a method factor for testing[32]. Therefore, we build a two-factor model in AMOS by adding a method factor as a global factor to the originally designed factors. If the model fit indices become much better after the addition of the method factor (e.g., CFI and TLI increase by more than 0.1 and RMSEA decrease by more than 0.05), it indicates that there is a serious common method bias[33].

Continuous variables are represented by (mean ± standard deviation), and categorical variables are represented by frequency and percentages. Independent samples t-test or one-way ANOVA was used to compare differences in the demographic characteristics. In the mediation effects analysis that followed, variables that showed significant differences in demographic characteristics were controlled for as covariates.

To verify the research hypotheses, the analyses were conducted in two stages. Firstly, Pearson's bivariate correlation analysis was used to analyze the relationship between variables. Secondly, the chain mediation effect was tested using Model 6 from the SPSS-PROCESS macro program. The PROCESS macro employs a stepwise approach for mediation testing, followed by a Bootstrap 95% confidence interval (CI) to determine the indirect effects based on 5000 bootstrapped samples. As a non-parametric resampling procedure, bootstrapping is considered the most powerful method for small samples because it is the least vulnerable to type 1 errors. If the 95% CI did not include 0, the effects were considered significant (P<0.05). The SPSS-PROCESS macro, developed by Andrew F. Hayes in 2013, has been widely validated in the literature for its robust analytical capabilities[34]. This program covers nearly a hundred mediation and moderation models and simplifies the analysis process, providing comprehensive results quickly. Its main advantage lies in the ability to perform bias-corrected, non-parametric percentile bootstrap tests and to provide the specific path coefficients for each mediator, along with the individual mediation effect test results for each mediator variable.” [Page 8, Line 205-224; Page 9, Line 225-236]

Results

13. The results section effectively addresses key points, but it is advisable to include information about each table below it.

Response: Thank you very much for your suggestion. We have added the relevant information in the notes of each table and figure to aid in understanding. We also sincerely apologize for the oversight of Table 4 in the previous version, which has now been included in full in the revised version. [see revised Page 11, Line 253-254, 266; Page 12, Line 283; Page 13, Line 285-288; Page 14, Line 301-308]

Discussion

14. (1) The discussion is well-structured, but some sentences could be more concise for better readability.

(2) The researchers should be ensuring consistent use of terms. For instance, “elderly patients with CHD” and “older adults” are used interchangeably. It might be helpful to stick to one term throughout the discussion.

(3) The explanation of the mediating roles of eHealth literacy and healthcare technology self-efficacy is good. However, the researcher might want to elaborate on how these mediators specifically influence the relationship between social support and technophobia. For instance, they can provide more examples or scenarios illustrating these mediating effects.

Response: We feel great thanks for your professional review work on our article. In response to the above three suggestions, we have thoroughly revised the discussion section. First, we carefully streamlined the sentences in the discussion to improve clarity. Second, we standardized the terminology for the study population, consistently referring to them as "elderly patients with coronary heart disease." However, in some instances, we simplified this to "elderly patients" to avoid redundancy. Finally, we re-explained the separate and chained mediating roles of eHealth literacy and healthcare technology self-efficacy, thereby enhancing the clarity of the discussion.

Here is the revised version:

“The results of this study showed that the technophobia score among elderly patients with CHD was 38.03±11.90, which was at a moderate level, consistent with reported by Peng et al.[26]. Before introducing the mediator variable, the effect value of social support on technophobia was -0.441. Additionally, after adding the mediator variable, the direct effect accounted for 38.5% (-0.257) of the total effect, confirming a negative correlation between social support and technophobia. The result was consistent with previous research, such as that by L. Li et al. which observed reduced technophobia in elderly cancer patients with higher levels of family support and social engagement[35]. These findings supported hypothesis 1. As older adults experience cognitive and physical decline, they are often challenged by age stereotypes and difficulties embracing novel technologies[36]. Elderly patients may experience psychological stress when confronted with emerging technologies. According to the buffer model in social support theory, social support can mitigate the impact of stressful events on individuals by either reducing the cognitive appraisal of stress or providing solutions to address specific issues[37]. Kwan et al. underscored the mental health benefits of social support and its role in preventing anxiety symptoms[21]. Elderly individuals with robust social support networks, including family members, peers and society can draw upon emotional and practical assistance when learning digital health technologies, thereby less fear of technologies, as evidenced by Lee et al.[20].

Mediation effect analysis found that eHealth literacy partially mediated between social support and technophobia, with its individual mediation effect accounting for 24.9% of the total indirect effect (validating hypothesis 2).This indicates that social support can directly influence technophobia in elderly patients with CHD while also exerting an indirect effect through eHealth literacy. This may be due to the fact that older patients with greater social support are more likely to have greater access to health information and resources, thereby avoiding the negative emotions caused by low eHealth literacy. When elderly patients with CHD feel supported by family, friends, and society, they are better equipped to face the challenges of new technology with a positive mindset and actively seek assistance[38]. Studies have shown that health knowledge seeking, contributing to health knowledge, and emotional support can all improve eHealth literacy[39]. A high level of eHealth literacy means that patients have the ability to use digital health technology proactively, resulting in less technophobia when engaging with technology[40].

Healthcare technology self-efficacy also played a partial mediating role between social support and technophobia, with its individual mediating effect accounting for 30.2% of the total indirect effect (validating hypothesis 3). The result was consistent with previous research, which found that individuals with higher healthcare technology self-efficacy have greater confidence in mastering the utilization of health technology, subsequently making them less prone to experiencing fear and avoidance[24]. Self-efficacy theory suggests that self-efficacy not only has an impact on individuals’ behaviors and decision-making but also has a direct effect on their psychological responses during activities[27]. Individuals with high self-efficacy tend to adopt a problem-focused coping style and respond to challenges and setbacks with greater confidence and resilience. When encouraged by family or friends, elderly patients with CHD gain confidence in overcoming difficulties and in trying new technologies, which fosters a high level of healthcare technology self-efficacy and, consequently, reduces their likelihood of experiencing technophobia[26].

This research found that eHealth literacy and healthcare technology self-efficacy jointly played a chain mediating role in the influence of social support on technophobia among elderly patients with CHD, with the mediating effect accounting for 44.9% of the total indirect effect(supporting hypothesis 4). That is, social support not only directly predicts technophobia in elderly patients with CHD but also through the chain effects of eHealth literacy and healthcare technology self-efficacy. Sequential pathways can explain this mediating effect. Specifically, social support provides elderly patients with CHD with the confidence to face difficulties and challenges, effectively alleviating psychological stress related to their illness[41]. This gives patients the confidence to recover and a willingness to access digital technologies such as the Internet. Additionally, social support can offer guidance and assistance in using technology, which helps to improve eHealth literacy[42]. Enhanced eHealth literacy leads to positive experiences with technology, strengthening patients' self-efficacy in using medical technology[43]. As a result, patients are more likely to use new technologies with confidence, reducing the likelihood of negative emotions or avoidance behaviors[26]. Research has shown that programs such as "technology feedback" and "intergenerational support" are effective in bridging the intergenerational gap by helping older people through their children or younger generations, improving their eHealth literacy and self-efficacy, and enabling them to integrate more smoothly into the digital society[20],[44]. ” [Page 14, Line 310-317; Page 15, Line 318-347; Page 16, Line 348-377; Page 17, Line 378-396;]

15. It is recommended to highlight the practical implications of the findings.

Response: Thank you very much for your suggestion. In the final part of the discussion section, we have provided a detailed addition regarding the implications of this study for clinical practice.

Here is the revised version: “According to the findings, to alleviate technophobia among elderly patients with CHD, who often rely on family and social support due to aging and illness, healthcare professionals can initiate interventions focusing on social support. Encouraging family members to assist elderly patients with CHD in actively using digital health technology can facilitate the implementation of family-centered cardiac rehabilitation programs. Relevant departments within hospitals or community healthcare centers can organize training sessions on technological skills, providing elderly CHD patients with spaces for learning and interaction. During medical visits, healthcare providers should offer technical support to prevent patients from feeling intimidated by technology, which might deter them from seeking medical care. eHealth literacy and healthcare technology self-efficacy are important mediums between social support and technophobia. Therefore, eHealth literacy should be emphasized when instructing older patients with CHD about digital health technology. Enhancing elderly patients' ability to use technology and the Internet and to recognize false information can empower them to make informed health decisions, such as adjusting their diet and exercising. Additionally, healthcare professionals should aim to boost elderly patients’ healthcare technology self-efficacy, encouraging them to engage in continuous self-improvement, which fosters confidence, independence, and effective disease management, ultimately improving health outcomes.” [Page 17, Line 397-407; Page 18, Line 408-412]
