# Supplementary material for: Social support and technophobia in older patients with coronary heart disease: The mediating roles of eHealth literacy and healthcare technology self-efficacy
Source: PLoS One. 2025 Jun 4;20(6):e0325202. doi: 10.1371/journal.pone.0325202 (PMC12136290; doi:10.1371/journal.pone.0325202)
Supplement: Attachment — Submitted filename: Response_to_Reviewers_auresp_2.docx [file pone.0325202.s004.docx]

March 9, 2025

Dear Prof. Seyedeh Yasamin Parvar,

RE: Manuscript ID PONE-D-24-40957R1

Thank you very much for giving us the opportunity to revise our manuscript entitled “Social support and technophobia in older patients with coronary heart disease: The mediating roles of eHealth literacy and healthcare technology self-efficacy”. We appreciate the valuable and constructive comments and suggestions from the editor, reviewers, and statisticians on this manuscript. Their insightful comments and suggestions have undoubtedly contributed to the improvement of our manuscript.

We have deliberately considered all comments and suggestions and have revised our manuscript accordingly. Please find our point-by-point responses in the response letter.

With the commitment and approval of all authors, we hereby submit our revised manuscript. We guarantee that neither the text nor the abstract of this paper has been published elsewhere in any language. We have no conflicts of interest to declare.

Thank you once again for your time, consideration, and the opportunity to submit our revised manuscript. We hope that the revised manuscript meets your requirements for publication in PLOS ONE.

Yours sincerely,

Aimin Wang

Institution: College of Nursing, Qingdao University

E-mail: wam@qdu.edu.cn

The point-by-point replies to all comments have been provided as follows:

**Journal requirements:**

1. Please review your reference list to ensure that it is complete and correct. If you have cited papers that have been retracted, please include the rationale for doing so in the manuscript text, or remove these references and replace them with relevant current references. Any changes to the reference list should be mentioned in the rebuttal letter that accompanies your revised manuscript. If you need to cite a retracted article, indicate the article’s retracted status in the References list and also include a citation and full reference for the retraction notice.

Response: Thank you for your request. We have added 4 references (Refs. 11, 31, 32, 48) because of changes to the article. We have carefully reviewed the reference list and found no errors or citations of retracted articles. However, we are open to any further feedback or specific areas that may need further refinement.

**Reviewer(s) comments:**

**Reviewer #2:**

1. Figure 1 quality should be enhaced. The meaning of the asterisks should be added to a figure legend.

Response: Thank you very much for your careful review. We sincerely apologize for this error. We have rechecked Table 1 and found that the p-values were already included, so no additional annotation was necessary. Therefore, we made the following revisions: removed the asterisks superscripted to the p-values and supplemented the missing data in the table. These changes have been completed in the revised manuscript. [Page 10-11, Line 258-259]

**Reviewer #3:**

1. Standardize terminology, such as consistently using "older adults" or "elderly patients with CHD" throughout the manuscript.

Response: Thank you very much for your suggestion. We have standardized the terminology for the study population. And for consistency with the title, we've changed it to “older patients with CHD” in the revised version.

2. Avoid redundancy in the discussion and simplify language for improved readability.

Response: Thank you very much for your suggestion. We have simplified the language of the discussion section in the revised version by, for example, removing redundant expressions and consolidating sentences with the same meaning.

Here is the revised version:

(1) “The result was consistent with previous research, which observed reduced technophobia in elderly cancer patients with higher levels of family support and social engagement[39].” [Page 15, Line 323-325]

(2) “Due to cognitive and physical decline, older patients with CHD may experience psychological stress when confronted with emerging technologies[40].” [Page 15, Line 325-328]

(3) “Older patients with CHD with robust social support networks can draw upon emotional and practical assistance when learning digital health technologies, thereby reducing fear of technologies, as evidenced by Lee et al.[21]. ” [Page 15, Line 332-335]

(4) “When older patients with CHD feel supported by family, friends, and society, they are better equipped to face the challenges of new technology[42]. Studies have shown that health knowledge seeking and emotional support can all improve eHealth literacy[43], and a high level of eHealth literacy can help reduce technophobia[44].” [Page 15, Line 342-346; Page 16, Line 347-348]

(5) “Encouragement from family and friends can enhance the self-efficacy of older patients with CHD, enabling them to face challenges with greater confidence and resilience, thereby reducing the occurrence of technophobia[27].” [Page 16, Line 356-362]

3. Strengthen the articulation of how this study fills gaps in existing research.

Response: Thank you very much for your comment. We have added an elaboration on how to fill research gaps in the last paragraph of the introduction section.

Here is the revised version: “The relationship between social support, eHealth literacy, and self-efficacy has also been established, particularly in the self-management of older patients with chronic diseases and their use of mobile health technologies[19, 30]. However, to our knowledge, the underlying relationship between these factors and technophobia remains unexplored in existing literature. According to the social ecosystem theory[31], the external environment can influence individual cognition and behavior, which in turn affects the psychological state of older adults when facing technology[27]. ” [Page 5, Line 120-126]

4. Provide more detailed clarification about the communities chosen for the study and their representativeness of the larger population.

Response: Thank you very much for this suggestion. We have added a detailed description of the recruitment area in the Participants section.

Here is the revised version: “Qingdao is an economically developed city in northern China with a population of about 10 million, of which the elderly account for 23.8% of the total population[32]. The four communities were randomly selected from each of Qingdao's four main municipal districts (Shinan, Shibei, Laoshan, Licang), and potential participants lived in both urban and rural communities.” [Page 5, Line139; Page 6, Line 140-143]

5. Offer more comprehensive suggestions for future study designs, including longitudinal approaches or more diverse sampling strategies.

Response: Thank you very much for this suggestion. We have added more specific recommendations in the Study Limitations section.

Here is the revised version:

(1) “Future studies should adopt an intervention or longitudinal approach to examine the real causal relationship.” [Page 18, Line 411-412]

(2) “Future studies should include participants from diverse regions and backgrounds through multi-centre, large-scale study designs to enhance the generalizability of findings.” [Page 18, Line 421-423]

(3) “Future studies should use more objective measures combined with qualitative, observational and experimental approaches to explore the interactions between social support, eHealth literacy, healthcare technology self-efficacy and technophobia.” [Page 18, Line 425-427]

6. Emphasize the practical meaning of coefficients and effect sizes beyond their statistical significance. Discuss the actionable significance of findings, such as how social support reduces technophobia by 44.9% through mediating factors.

Response: We feel great thanks for your professional review work on our article. In this study, the direct effect accounting for 38.5% indicates that even without considering the mediating variables (such as eHealth literacy and healthcare technology self-efficacy), social support alone can significantly reduce technophobia. The larger proportion of the indirect effect compared to the direct effect suggests that social support primarily influences technophobia through mediating variables. Within the indirect effects, the chain mediation effect of eHealth literacy and healthcare technology self-efficacy represents the most significant pathway, contributing nearly half of the total indirect effect. Therefore, in the Discussion section, we explained the implications of these effect proportions and emphasized their relevance in clinical practice.

Here is the revised version:

(1) “Additionally, after adding the mediator variable, the direct effect accounted for 38.5% (-0.257) of the total effect, even without considering the mediator variable, social support can significantly reduce technophobia.” [Page 15, Line 320-323]

(2) “This research found that eHealth literacy and healthcare technology self-efficacy jointly played a chain mediating role in the influence of social support on technophobia among elderly older patients with CHD, with the mediating effect accounting for 44.9% of the total indirect effect (supporting hypothesis 4). This suggests that social support influencing technophobia through the chain-mediated effects of eHealth literacy and healthcare technology self-efficacy is the predominant indirect pathway, contributing nearly half of the total indirect effect.” [Page 16, Line 363-370]

(3) “According to the findings, to alleviate technophobia among elderly older patients with CHD, the relevant departments of hospitals and communities should establish a comprehensive social support system for them. [Page 17, Line 382-385]

(4) “In addition, the findings suggest that the chain-mediated effect of eHealth literacy and healthcare self-efficacy is the core mechanism. When reducing technophobia through social support, efforts should focus on these two aspects.” [Page 17, Line 396-398]

7. Provide concrete examples of how social support, eHealth literacy, and self-efficacy can be enhanced in clinical settings.

Response: Thank you very much for your suggestion. We have added examples of how social support, eHealth literacy, and healthcare technology self-efficacy can be improved in clinical settings in the final paragraph of the discussion section.

Here is the revised version: “According to the findings, to alleviate technophobia among older patients with CHD, the relevant departments of hospitals and communities should establish a comprehensive social support system for them. Healthcare professionals should encourage intergenerational interaction between patients and family members , especially with younger generations, to facilitate digital technology communication[47]. Peer-based technology support groups should be established for older patients with CHD, facilitating experience sharing through both offline activities and online communities[48]. Community healthcare centers can organize training sessions on technological skills, providing patients with spaces for learning and interaction[48]. In addition, the findings suggest that the chain-mediated effect of eHealth literacy and healthcare self-efficacy is the core mechanism. When reducing technophobia through social support, efforts should focus on these two aspects. Family members and professionals should prioritize improving patients’ basic technical competence and critical thinking skills to identify misinformation[21]. Instructors should provide immediate encouragement after patients master each technical skill to enhance self-efficacy. For anxiety management, instructors can guide patients to adopt positive self-affirmations during technology use.” [Page 17, Line 382-402]

8. Include more detailed explanations of statistical terms and findings to aid readers. For instance, expand on the interpretation of mediation effects and bootstrap confidence intervals.

Response: Thank you very much for your advice. We have added explanations of chained mediation effect and the 95% confidence interval in the revised version.

Here is the revised version:

(1) “Secondly, the chain mediation effect was tested using Model 6 from the SPSS-PROCESS macro program, which refers to the indirect effect in a causal pathway where the influence of an independent variable (X) on a dependent variable (Y) is transmitted sequentially through multiple mediators (e.g., M₁, M₂) in a specified order.” [Page 9, Line 226-230]

(2) “The 95% CI is a statistical range used to estimate the plausible values of mediation effect. If the study were repeated 100 times, approximately 95 of the calculated intervals would contain the true parameter value.” [Page 9, Line 233-235]

**Reviewer #4:**

1. There are two objectives for this article, one is a sentence and the other is a phrase in need of unification and revision.

Response: Thank you very much for your advice. Your suggestion was so helpful that we have changed the first sentences of the abstract in the revised version.

Here is the revised version: “The purpose of this study was to explore the relationship between social support, eHealth literacy, healthcare technology self-efficacy, and technophobia. It also analyzed the mediating effect of eHealth literacy and healthcare technology self-efficacy between social support and technophobia.” [Page 2, Line 28-30]

2. On page 4, there appears to be an absence of a logical connection between the first and second paragraphs. It would be preferable to refer to social support as one of the supporting factors at the end of the first paragraph.

Response: Thank you very much for your suggestion. Based on your guidance, we introduced social support as one of the supporting factors at the end of the third paragraph of the introduction. And we also modified the first sentence of the fourth paragraph to make it articulate.

Here is the revised version:

(1) “As an important coping resource, social support is considered a key external protective factor[18, 19].” [Page 4, Line 91-92]

(2) “Social support has been found to influence technology adoption and utilization among individuals, and there is a significant negative correlation between social support and technophobia in older adults[20].” [Page 4, Line 93-95]

3. I would like to suggest that in the introduction, you could perhaps use the following reference to further emphasize the importance of digital health, and specifically telehealth, in the management of CHD: Hayavi-Haghighi MH, Gharibzade A, Choobin N, Ansarifard H. Applications and outcomes of implementing telemedicine for hypertension management in COVID-19 pandemic: A systematic review. PLoS One. 2024 Aug 1;19(8):e0306347. doi: 10.1371/journal.pone.0306347. PMID: 39088489; PMCID: PMC11293715

Response: Thank you very much for your suggestion, it is very helpful. We have incorporated the perspective from this literature into the Introduction section and included a citation to the source in the revised version.

Here is the revised version: “Most patients and healthcare professionals believe that digital health technologies can provide convenient and effective medical services[11].” [Page 3, Line 74-75]

4. On page 4, paragraph 2, delete the “in conclusion” from the beginning of the paragraph.

Response: Thank you very much for this suggestion. We have deleted “in conclusion” in the revised version.

Here is the revised version: “Previous studies have shown that social support, eHealth literacy, and healthcare technology self-efficacy play an important role in influencing technophobia in older patients with CHD.” [Page 5, Line 118-120]

5. An inaccuracy was observed in the provision of social support cut points. As with Part technophobia, the provision of complete information regarding this matter, in addition to the utilization of the Likert scoring method, is imperative.

Response: Thank you very much for this suggestion. We have supplemented the scoring of the Social Support Rating Scale and corrected errors in the cut-off points.

Here is the revised version: “The Social Support Rating Scale (SSRS) which was compiled by Xiao in 1994[34], was used to measure social support. The scale includes 10 items and 3 dimensions: subjective support, objective support, and utilization of support. Items 1-4 & 8-10: Select one option per item (1-4 points). Item 5: A-D options (4-point scale: 1=none to 4=full support). Items 6-7: 0 points without sources; score = number of sources listed. The total score on the scale ranges from 12 to 66, with higher scores representing more social support. A total score of 12–22 indicates a low level of social support, 23–44 indicates a medium level of social support, and 45–66 indicates a high level of social support. The SSRS has good reliability and validity with Cronbach’s α of 0.89 to 0.94. The Cronbach’s α of this scale in this study was 0.87.” [Page 6, Line 166-167; Page 7, Line 168-174]

6. There is no proper caption for Table 4 in the text.

Response: Thank you very much for your suggestion. We have modified the title of Table 4.

Here is the revised version: “Table 4. The mediating effect of eHealth literacy and healthcare technology self-efficacy between social support and technophobia.” [Page 14, Line 307-309]

7. The discussion is written in a coherent and argumentative style, but there is a need for greater citation of sources, particularly in the final paragraph of the discussion (p. 16).

Response: We feel great thanks for your professional review work on our article. We have revised the final paragraph of the Discussion section based on other suggestions and added the relevant references as per your guidance.

Here is the revised version: “According to the findings, to alleviate technophobia among older patients with CHD, the relevant departments of hospitals and communities should establish a comprehensive social support system for them. Healthcare professionals should encourage intergenerational interaction between patients and family members , especially with younger generations, to facilitate digital technology communication[47]. Peer-based technology support groups should be established for older patients with CHD, facilitating experience sharing through both offline activities and online communities[48]. Community healthcare centers can organize training sessions on technological skills, providing patients with spaces for learning and interaction[48]. In addition, the findings suggest that the chain-mediated effect of eHealth literacy and healthcare self-efficacy is the core mechanism. When reducing technophobia through social support, efforts should focus on these two aspects. Family members and professionals should prioritize improving patients’ basic technical competence and critical thinking skills to identify misinformation[21]. Instructors should provide immediate encouragement after patients master each technical skill to enhance self-efficacy. For anxiety management, instructors can guide patients to adopt positive self-affirmations during technology use.” [Page 17, Line 382-402]

**Reviewer #5:**

1. Clarity of Mediating Effects: While the mediating roles of eHealth literacy and self-efficacy are statistically supported, further elaboration on the practical implications of these findings would enhance the manuscript’s impact.

Response: We feel great thanks for your professional review work on our article. In this study, the direct effect accounting for 38.5% indicates that even without considering the mediating variables (such as eHealth literacy and self-efficacy), social support alone can significantly reduce technophobia. The larger proportion of the indirect effect compared to the direct effect suggests that social support primarily influences technophobia through mediating variables. Within the indirect effects, the chain mediation effect of eHealth literacy and healthcare technology self-efficacy represents the most significant pathway, contributing nearly half of the total indirect effect. Therefore, in the Discussion section, we explained the implications of these effect proportions and emphasized their relevance in clinical practice.

Here is the revised version:

(1) “Additionally, after adding the mediator variable, the direct effect accounted for 38.5% (-0.257) of the total effect, even without considering the mediator variable, social support can significantly reduce technophobia.” [Page 15, Line 320-323]

(2) “This research found that eHealth literacy and healthcare technology self-efficacy jointly played a chain mediating role in the influence of social support on technophobia among elderly older patients with CHD, with the mediating effect accounting for 44.9% of the total indirect effect (supporting hypothesis 4). This suggests that social support influencing technophobia through the chain-mediated effects of eHealth literacy and healthcare technology self-efficacy is the predominant indirect pathway, contributing nearly half of the total indirect effect.” [Page 16, Line 363-370]

(3) “According to the findings, to alleviate technophobia among elderly older patients with CHD, the relevant departments of hospitals and communities should establish a comprehensive social support system for them. [Page 17, Line 382-385]

(4) “In addition, the findings suggest that the chain-mediated effect of eHealth literacy and healthcare self-efficacy is the core mechanism. When reducing technophobia through social support, efforts should focus on these two aspects.” [Page 17, Line 396-398]

2. Generalizability of Results: The study's sample is limited to a specific province in China. Addressing potential cultural or regional biases and suggesting broader applications would improve the manuscript's robustness.

Response: Thank you very much for your advice. To address this issue, we first supplemented detailed information about the recruitment regions in the Participants section to ensure the sample has a certain level of representativeness. Second, in the Study Limitations section, we proposed broader application recommendations to mitigate potential cultural or regional biases.

Here is the revised version:

(1) “Qingdao is an economically developed city in northern China with a population of about 10 million, of which the elderly account for 23.8% of the total population[32]. The four communities were randomly selected from each of Qingdao's four main municipal districts (Shinan, Shibei, Laoshan, Licang), and potential participants lived in both urban and rural communities.” [Page 5, Line139; Page 6, Line 140-143]

(2) “Future studies should include participants from diverse regions and backgrounds through multi-centre, large-scale study designs to enhance the generalizability of findings.” [Page 18, Line 421-423]

3. Language and Grammar: The manuscript's language is generally clear, but minor grammatical refinements would improve readability.

Response: We tried our best to improve the manuscript and made some changes to the manuscript. These changes will not influence the content and framework of the paper. And here we did not list the changes but marked in red in the revised paper. We hope that the correction will meet with approval.
